# Soluble Epoxide Hydrolase Inhibitor TPPU Alleviates Nab-Paclitaxel-Induced Peripheral Neuropathic Pain via Suppressing NF-*κ*B Signalling in the Spinal Cord of a Rat

**DOI:** 10.1155/2023/9058774

**Published:** 2023-02-08

**Authors:** Xing Wei, Lijun Jia, Yaqing Zhou, Weimiao Li, Changyou Shan, Shuqun Zhang, Yonglin Zhao

**Affiliations:** ^1^Department of Gynaecology and Obstetrics, The Second Affiliated Hospital of Xi'an Jiaotong University, Xi'an 710004, China; ^2^Department of Oncology, The Second Affiliated Hospital of Xi'an Jiaotong University, No. 157 Xiwu Road, Xi'an 710004, China

## Abstract

**Objective:**

Paclitaxel-induced peripheral neuropathy (PIPN) is a debilitating and difficult-to-treat side effect of paclitaxel. Soluble epoxide hydrolase (sEH) can rapidly metabolize the endogenous anti-inflammatory mediators' epoxyeicosatrienoic acids (EETs) to dihydroxyeicosatrienoic acids. This study aimed to assess whether the sEH inhibitor N-(1-(1-oxopropy)-4-piperidinyl]-N′-(trifluoromethoxy) phenyl)-urea (TPPU) plays a critical role in PIPN of rats and provides a new target for treatment.

**Methods:**

A Sprague–Dawley male rat model of PIPN induced by nab-paclitaxel was established. Rats were randomly divided into a control group, nab-paclitaxel group, and nab-paclitaxel + TPPU (sEH inhibitor) group, with 36 rats in each group. The effects of the sEH inhibitor TPPU on behavioural assays, apoptosis, glial activation, axonal injury, microstructure, and permeability of the blood-spinal cord barrier were detected, and the underlying mechanisms were explored by examining the expression of NF-*κ*B signalling pathways, inflammatory cytokines, and oxidative stress.

**Results:**

The results showed that the mechanical and thermal pain thresholds of rats were decreased after nab-paclitaxel treatment, accompanied by an increased expression of axonal injury-related proteins, enhanced cell apoptosis, aggravated destruction of vascular permeability, intense glial responses, and elevated inflammatory cytokines and oxidative stress in the L4-L6 spinal cord. TPPU restored the mechanical and thermal thresholds, decreased cell apoptosis, alleviated axonal injury and glial responses, and protected vascular permeability by increasing the expression of tight junction proteins. TPPU relieved PIPN by inhibiting the activation of the sEH and NF-*κ*B signalling pathways by decreasing the levels of inflammatory cytokines and oxidative stress.

**Conclusion:**

These findings support a role for sEH in PIPN and suggest that the inhibition of sEH represents a potential new therapeutic target for PIPN.

## 1. Introduction

Paclitaxel-induced neuropathic pain (PINP) is a common and difficult-to-treat side effect that can persist for months to years after paclitaxel treatment. PINP could result in significant symptoms such as burning pain and allodynia and affect the quality of life of patients [1]. A previous study found that the mechanism of PINP might be related to neuroinflammation, mitochondrial dysfunction, and disordered cation channels [2]. Although several drugs, including anti-inflammatory drugs, opioids, and antidepressants, are recommended to treat PINP, they cannot obtain satisfactory results in clinical trials [2]. The concrete mechanism of PINP needs to be further studied.

Epoxyeicosatrienoic acids (EETs) are important angiogenic mediators and could be degraded by soluble epoxide hydrolase (sEH) [3]. EETs have been shown to exert beneficial effects on neuropathic pain [3]. A previous study found that EETs could prevent continued nerve damage by decreasing the levels of inflammatory factors and promoting nerve regeneration [3]. EET actions also provide superior pain relief involving both the endogenous opioid and GABAergic systems [3]. Genetic deletion or pharmacologic inhibition of sEH have been demonstrated to greatly help in maintaining or elevating EET levels *in vivo* to achieve beneficial effects in several disease models [4]. Nonetheless, the role of sEH inhibitors in PINP is still unknown.

The current study aimed to investigate whether sEH inhibition could alleviate PIPN by influencing the integrity of the blood-spinal cord barrier (BSCB). To simulate the PIPN of humans, a rat model of PIPN was established by nab-paclitaxel. We detected the effects of the sEH inhibitor on behavioural assays, apoptosis, glial activation, axonal injury, microstructure, and permeability of the BSCB. Our results showed that the protective effects of the sEH inhibitor were associated with decreases in levels of inflammatory factors and oxidative stress, suggesting that the sEH inhibitor may be a prospective target in the therapy of PINP.

## 2. Materials and Methods

### 2.1. Experimental Animals and Ethical Approval

Male SD rats were obtained from the Experimental Animal Center of Xi'an Jiaotong University and the number of the animal license is SCXK (Shaanxi) 2006-001. All the rats were 8–10 weeks old and weighed 250–300 g. The Biomedical Ethics Committee for Animal Experiments of Shaanxi Province (China, 2021-0145) approved this investigation. All procedures were carried out in accordance with the Guidelines for the Care and Use of Laboratory Animals. The rats had free access to food and water and housed 5 per cage. Rats were raised at an ambient temperature of 22 ± 1°C on a 12-h light/dark cycle.

### 2.2. Foundation of the Rat Model of PIPN

**Figure 1:**
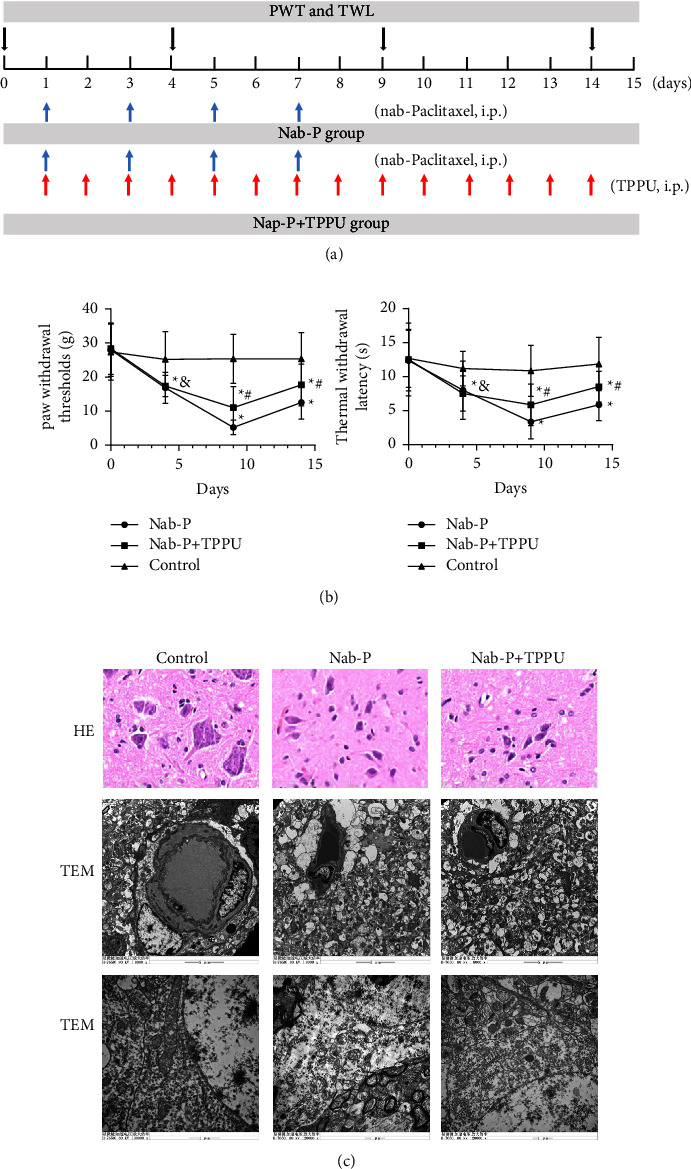
TPPU alleviated nab-paclitaxel-induced mechanical, heat hypersensitivity, and pathological changes. (a) Schematic diagram of the experimental procedure. Nab-P, nab-paclitaxel; TPPU, N-(1-(1-oxopropy)-4-piperidinyl]-N′-(trifluoromethoxy)phenyl)-urea; i.p., intraperitoneally; PWT, paw withdrawal mechanical threshold; TWL, thermal withdrawal latency. Blue arrows indicated the timepoint when nab-P was treated on rats, while red arrows indicated when TPPU was treated on rats. (b) PWT and TML of each group (*n* = 6) at d0, d4, d9, and d14. ^*∗*^*P* < 0.05 compared to the control group, ^#^*P* < 0.05 compared to the nab-P group, and ^&^P > 0.05 compared with the nab-P group. (c) H and E staining (scale bar = 100 *μ*m) and TEM (scale bar = 5 *μ*m and 1 *μ*m) were used to detect the pathological changes in the spinal cord of a rat.

In this study, to simulate a paclitaxel-induced neuropathic pain model, nab-paclitaxel (Shi Yao Group Ou Yi Pharmaceutical Co., Ltd., Hebei Province, China) was dissolved in saline. Rats were intraperitoneally injected with nab-paclitaxel on four alternate days (2.47 mg/kg on days 1, 3, 5, and 7) (Figure 1(a)) [5, 6]. Rats were given the nab-paclitaxel with a final cumulative dose of 9.9 mg/kg. The same dose of saline was given to the control rats. The rats were fed at a temperature of 22 ± 2°C after treatment until euthanasia. No rats died during the experiment. Rats were euthanized by intraperitoneally injecting 5% (w/v) pentobarbital sodium.

### 2.3. Groups and Drug Administration

Sample size estimation was calculated by a statistical power analysis. A one-way ANOVA with three groups was used with the assumption that *α* = 0.05 and power = 0.80. The suggested sample size was *n* = 36 per group (6 rats for pathology; 6 rats for TEM; 6 rats for western blot; 6 rats for ELISA and oxidative stress detection; 6 rats for Evans blue detection; 6 rats for spinal cord water content). By using a randomized digital table, the total 108 rats (sample size was decided according to a previous study) were randomized into three groups [7, 8]: (a) 36 rats in the control group; (b) 36 rats in the nab-paclitaxel (Nab-P) group, which was treated with nab-paclitaxel for four alternate days (days 1, 3, 5, and 7); and (c) 36 rats in the nab-paclitaxel + N-(1-(1-oxopropyl)-4-piperidinyl)-N′-(4-(trifluoromethoxy)phenyl)-urea (TPPU, sEH inhibitor) (nab-P + TPPU) group. Rats in the nab-P + TPPU group were treated with nab-paclitaxel in the same manner as the nab-P group and TPPU. In this group, TPPU (Merck KGaA, Darmstadt, Germany) was initially dissolved in 0.1% dimethyl sulfoxide (DMSO) as a stock solution and diluted to 1 mM as the working solution before each study [7, 8]. TPPU (3 mg/kg/day) was intraperitoneally injected on 14 consecutive days using a microinjection syringe (Figure 1(a)) [7, 8]. All rats were sacrificed on day 15 after the first nab-paclitaxel treatment. Mechanical hyperpathia and heat hypersensitivity were tested on 4, 9, and 14 days after the first treatment of nab-paclitaxel, respectively (Figure 1(a)). The order of treatments and measurements is randomized. Apart from the conductor, none was aware of the group allocation at the different stages of the experiment.

### 2.4. Behavioural Assays

The paw withdrawal threshold (PWT) and thermal withdrawal latency (TWL) were used to perform behavioural assays. PWT and TWL were tested at 0, 4, 9, and 14 days after the first nab-paclitaxel treatment at 1 day (Figure 1(a)). Calibrated von Frey filaments (Stoelting, WoodDale, USA) were applied to measure PWT after acclimation for 15 minutes [9]. Rat feet were stimulated with von Frey filaments, ranging from 2 to 26 × g bending force, and the minimum stimulus intensity (g) that resulted in feet shrinkage was recorded. Each irritation was consecutive for 5 s, and the interval between two irritation is 20 s. A plantar tester and an infrared radiant heat stimulus generator (Ugo Basile, Varese, Italy) were used to test the TWL of the hind paws [9]. A radiant heat source was used to focus on the hindlimb plantar. A foot shrinkage occurred as soon as the rats suffered pain. The delay time (s) of paw withdrawal was considered TWL [9]. All rats were tested 4 times and the timepiece between tests is 5 min.

### 2.5. Haematoxylin-Eosin Staining

The L4-L6 spinal cord was excised from the rat following transcardial perfusion with saline followed by 4% paraformaldehyde. They were sectioned longitudinally into 5 *μ*m sections before staining. Haematoxylin was used to stain tissue sections for 2.5 min and eosin for 16 s. Then, sections were obtained by dehydration, hyalinization, and fixation. Sections were observed under a high-power light microscope.

### 2.6. Immunoblot

The L4-L6 spinal cord was homogenized in a protease inhibitor lysis buffer, and the supernatants were collected. A bicinchoninic acid protein assay kit was used to determine the protein concentration. A total of 30–50 *μ*g of protein was separated using sodium dodecyl sulfate-polyacrylamide gel electrophoresis. The protein was then transferred to polyvinylidene difluoride membranes. Membranes were blocked and incubated overnight at 4°C with primary antibodies against sEH (1 : 1000, Cayman, Chemical, Ann Arbor, MI, USA), NF-*κ*B (1 : 1000, Cell Signaling Technology (CST), Danvers, MA, USA), and *β*-actin (1 : 1000, CST). A secondary antibody was used to incubate membranes for 1 hour. Immunoreactive proteins were detected by enhanced chemiluminescence. A luminescent image analyser was used to acquire images. Quantification of the western blot bands was performed using Image J software. The results were normalized to *β*-actin.

### 2.7. Terminal Deoxynucleotidyl Transferase-MediateddUTP-Biotin Nick End Labelling (TUNEL) Assay

Cell apoptosis was confirmed by a DeadEnd Fluorometric TUNEL System (Promega, Madison, Wisconsin, USA) using paraffin sections. Briefly, paraffin sections of L4-L6 spinal cords were deparaffinized, rehydrated, and digested with proteinase K. Sections were then covered with an equilibration buffer and incubated with TdT Labelling Reaction Mixture in a humid atmosphere. After that, the nuclei were stained with DAPI. Six fields were randomly selected for the count of apoptotic cells at 400x magnification.

### 2.8. Immunohistochemistry

After anaesthesia, the L4-6 spinal cord was harvested after prefixation performed by perfusion with saline followed by 4% paraformaldehyde. Spinal cords were dehydrated, embedded in paraffin, and sliced. Slices were deparaffinized and rehydrated in graded ethanol solutions. After retrieving antigens, hydrogen peroxide was used to block endogenous peroxidase. Slices were then incubated with primary antibodies, including glial fibrillary acidic protein (GFAP) (1 : 500, CST), Iba-1 (1 : 400, Wako, Tokyo, Japan), *β*-APP (1 : 400, Abcam, Cambridge, UK), neurofilament light chain (NF-L, 1 : 200, CST), neurofilament heavy chain (NF-H, 1 : 200, CST), and neurofilament medium chain (NF-M, 1 : 200, CST). Then, HRP-conjugated secondary antibodies were used to incubate slices. Slices were further stained by DAB. Immunohistochemical scores (IHS) were used to evaluate immunohistochemical results. The quantity scores multiplied by staining intensity scores equals IHS. The quantity scores ranged from 0–4 as follows: (1) no cell staining, 0; 1–10%, 1; 11–50%, 2; 51–80%, 3; and 81–100%, 4. The staining intensity scores ranged from 0–3 as follows: negative, 0; weak staining, 1; moderate staining, 2; and strong staining, 3. Theoretically, the scores ranged from 0 to 12 [10]. An experienced pathologist who was blinded to the experimental conditions was commissioned to detect the immunohistochemically stained sections.

### 2.9. Transmission Electron Microscopy

The ultrastructural changes were examined by transmission electron microscopy (TEM) in the L4-L6 spinal cords of rats. After being cut into 1 mm^3^ sections, spinal cords were fixed in glutaraldehyde (2.5%) overnight. Then, an ethanol and acetone gradient series was used to dehydrate the samples. Next, the tissue was soaked in linoleate at 60°C overnight. Ultramicrotome was used to cut slices after methylene blue staining. Finally, TEM (H-7650, Hitachi, Tokyo, Japan) was used to observe the spinal cord ultrastructure at 20,000x and 8,000x magnification.

### 2.10. Enzyme-Linked Immunosorbent Assay (ELISA)

The obtained L4-L6 spinal cord tissue was dissociated, homogenized, and then centrifuged. The concentrations of proinflammatory factors (interleukin-1*β* (IL-1*β*), interleukin-6 (IL-6), and tumour necrosis factor-*α* (TNF-*α*)) and anti-inflammatory factors (interleukin-4 (IL-4) and interleukin-10 (IL-10)) were detected by commercial ELISA kits. The absorbance value at 450 nm was measured. Data (pg protein) were normalized to mg of total protein.

### 2.11. Immunofluorescence Staining

The L4-L6 spinal cord was removed, fixed in 4% paraformaldehyde, and then embedded in paraffin. As with the procedure of immunohistochemistry, sections were then incubated with goat polyclonal anti-ZO-1 (1 : 200, Abcam) overnight at 4°C. The fluorochrome-conjugated secondary antibody was used to incubate sections. A fluorescence microscope was used to observe the stained slices.

### 2.12. Vascular Permeability Assay

The vascular permeability of the spinal cord was assessed by Evans blue. Evans blue (2%, 4 mL/kg of body weight, Sigma-Aldrich, St. Louis, MO, USA) was injected intravenously via the tail vein. After 1 h, rats were perfused with normal saline. L4-L6 of the spinal cord was quickly homogenized. The obtained proteins were precipitated with 50% trichloroacetic acid overnight. The supernatant was obtained after centrifugation. The wavelength is set to 610 nm in order to examine absorbance. The content of Evans blue was presented as micrograms per Gram of spinal cord tissues.

### 2.13. Evaluation of Spinal Cord Oedema

The wet-dry method was employed to assess spinal cord water content. Briefly, the L4-L6 spinal cord wet weight was measured. An oven was used to dry the spinal cord at 105°C for 72 h. The dry weight was measured. Spinal cord water content (%) was calculated by using the formula: (wet weight − dry weight)/wet weight × 100% [11].

### 2.14. Oxidative Stress Marker Measurement

The L4-L6 spinal cords were prepared, homogenized, and centrifuged. After protein quantification, tissue lysates were analysed to detect the levels and activities of malondialdehyde (MDA), superoxide dismutase (SOD), glutathione peroxidase (GSH), and catalase (CAT) according to procedures. The concentrations were calculated with reference to standard curves.

### 2.15. Statistical Analysis

The results are expressed as the mean ± standard deviation (SD). Statistical analysis was performed by SPSS 18.0 (SPSS, Chicago, IL, USA). One-way analysis of variance (ANOVA) was used to analyse data in more than 2 groups, followed by LSD (L) to conduct a post-hoc test. A *P* value less than 0.05 was considered statistically significant. Shapiro–Wilk's method is recommended for the normality test. A normal distribution of all the data is shown.

## 3. Results

### 3.1. Effects of the sEH Inhibitor on PINP and Pathological Changes in the Rat's Spinal Cord

The PWT and TWL were lower in the nab-P group at d4, d9, and d14 (*P* all <0.001) in comparison with the control group. Compared to the nab-P group at d9 and d14, the PWT and TWL were significantly increased in the Nab-P + TPPU group (*P* all <0.001), while there was no significant difference between these two groups at d4 (*P* > 0.05) (Figure 1(b)). The histopathological features and pathological changes in the rat spinal cord were evaluated. The cell morphology was normal in the control group, while neuronal pyknosis, contortion, and variant were detected in the nab-P group. Compared to the nab-P group, the histopathological lesions were alleviated in the Nab-P + TPPU group (Figure 1(c)). TEM images showed that the structure of mitochondria was integrated and that the structures around the microvessels were intact. No interstices, oedema, or distortion were observed. In the nab-P group, the structures around the microvessels were destroyed with large interstices and oedema. Mitochondria vacuoles, fracture, swelling, and disordered sparse cristae were observed. In the nab-P + TPPU group, the damage to microvessels and mitochondria was mitigated, with fewer interstices, vacuoles, fractures, and swelling than in the Nab-P group (Figure 1(c)).

### 3.2. Inhibition of sEH Relieved the Axonal Injury Induced by Nab-Paclitaxel in the Spinal Cord

**Figure 2:**
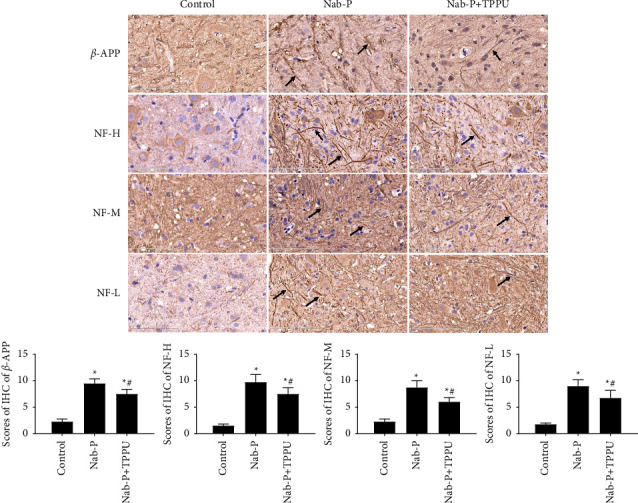
Inhibition of sEH-protected axons from nab-paclitaxel treatment. Pathological changes in axons were evaluated by immunohistochemistry of NF-L, NF-M, NF-H, and *β*-APP. Scale bars = 100 *μ*m. *n* = 6; ^*∗*^*P* < 0.05 compared to the control group, ^#^*P* < 0.05 compared to the nab-P group. Black arrows indicate the NF-L, NF-M, NF-H, and *β*-APP positive cells, respectively.

In immunostaining slices, NF-L, NF-M, NF-H, and *β*-APP were barely observed in the control group. The expression levels of NF-L, NF-M, NF-H, and *β*-APP were higher in the nab-P group (*P* all <0.001) in comparison with the control group. Compared with the nab-P group, the expression levels of NF-L, NF-M, NF-H, and *β*-APP were lower in the nab-P + TPPU group (Figure 2) (*P* all <0.001).

### 3.3. sEH Inhibition Alleviated Apoptosis and Glial Responses after Nab-Paclitaxel Treatment

**Figure 3:**
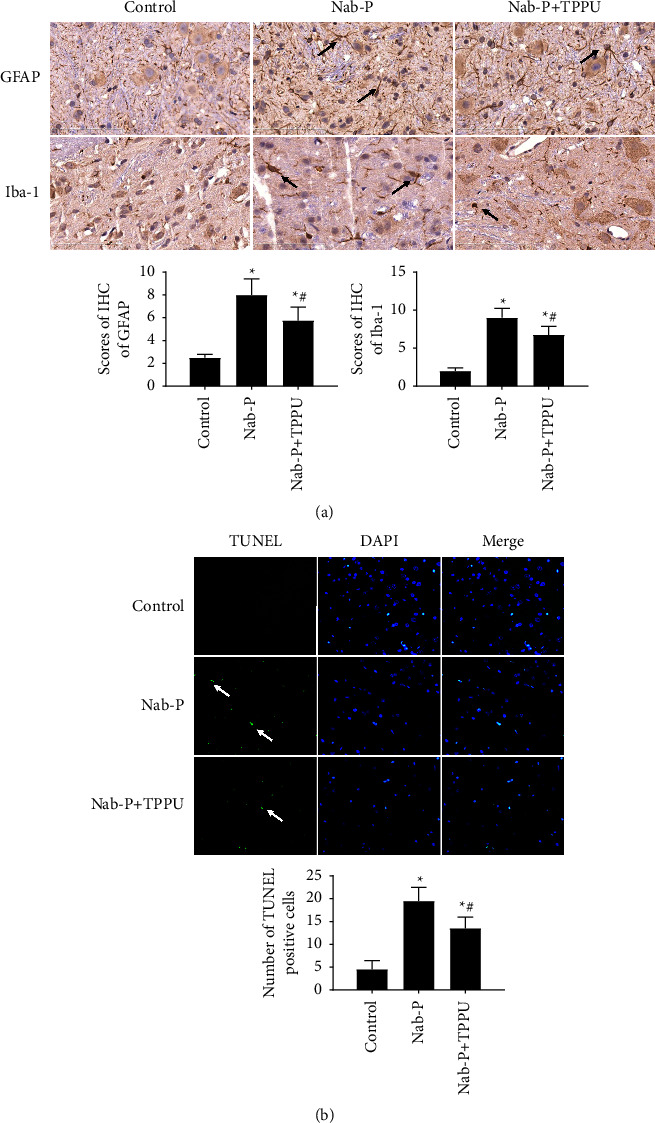
Inhibition of sEH significantly attenuates glial response and apoptosis after nab-paclitaxel treatment. (a) Immunohistochemistry was used to test the expression of GFAP and Iba-1 in the spinal cord of rats. Scale bars = 100 *μ*m. Black arrows indicate the GFAP and Iba-1 positive cells, respectively. (b) TUNEL assay was used to detect apoptotic cells. Stained green was considered as TUNEL-positive cells, and DAPI was used to stain the nuclei (blue) (×40 magnification). White arrows indicate the apoptotic cells. *n* = 6; ^*∗*^*P* < 0.05 compared with the control group, ^#^*P* < 0.05 compared to the nab-P group.

The expression of GFAP and Iba-1 is considered a marker of the glial response. The expression of GFAP and Iba-1 was significantly higher in the nab-P group (*P*=0.005, *P*=0.004, respectively) in comparison with the control group. Compared with the nab-P group, the expression of GFAP and Iba-1 was lower in the nab-P + TPPU group (Figure 3(a)) (*P*=0.005, *P*=0.006, respectively). TUNEL-positive cells were barely observed in the control group. The number of TUNEL-positive cells was higher in the nab-P group (*P*=0.006, *P*=0.008, respectively) in comparison with the control group. Compared with the nab-P group, the number of apoptotic cells was lower in the nab-P + TPPU group (Figure 3(b)) (*P*=0.007, *P*=0.007, respectively).

### 3.4. TPPU Ameliorated Vascular Permeability after Nab-Paclitaxel Treatment

**Figure 4:**
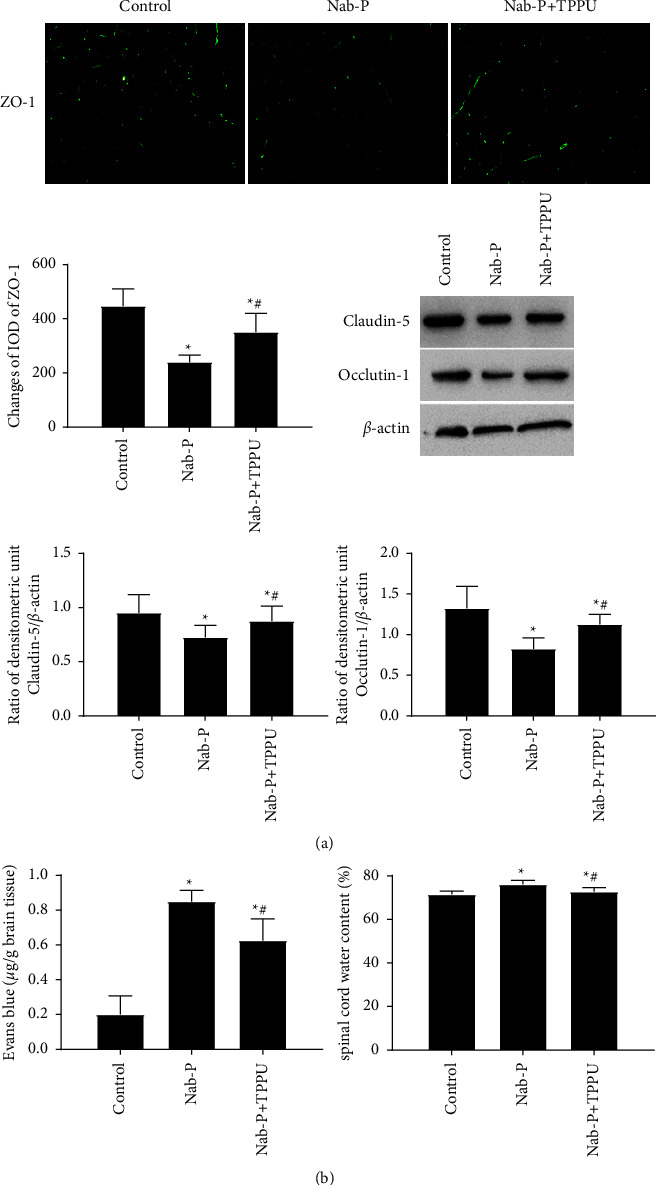
The effect of the sEH inhibitor TPPU on vascular permeability in the spinal cord after nab-paclitaxel administration. (a) The expressions of ZO-1, claudin-5, and occludin-1 were assessed by immunofluorescence staining and western blotting analysis. (b) The bar graphs show the statistical results of the Evans blue and spinal cord water content. *n* = 6; ^*∗*^*P* < 0.05, compared with the control group; ^#^*P* < 0.05, compared with the nab-P group.

The expressions of ZO-1, claudin-5, and occludin-1 were decreased in the nab-P group (*P*=0.002, *P*=0.004, and *P*=0.005, respectively) in comparison with the control group. sEH inhibition by TPPU significantly upregulated the expression of ZO-1, claudin-5 and occludin-1 compared to the nab-P group (Figure 4(a)) (*P*=0.003, *P*=0.004, and *P*=0.006, respectively). Little Evans blue diffusion was detected in the control group, while the level of Evans blue was significantly increased in the Nab-P group, and the content of Evans blue was decreased in the nab-P + TPPU group compared to the Nab-P group (*P* all <0.001). Compared with the control group, the oedema of the spinal cord assessed by water content was significantly higher in the nab-P group. The water content in the nab-P + TPPU group was decreased than that in the nab-P group (Figure 4(b)) (*P* all <0.001).

### 3.5. The Neuroprotective Effects of TPPU Were Related to the sEH/NF-*κ*B Pathway and Downstream Inflammatory Cytokines and Oxidative Stress Levels

**Figure 5:**
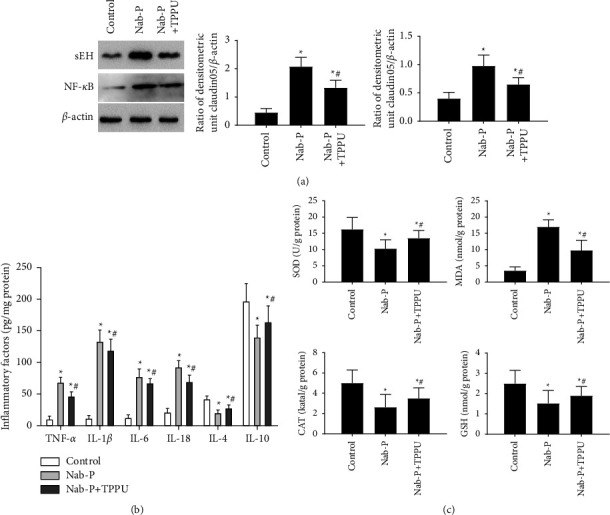
sEH/NF-*κ*B pathway-induced inflammatory factors and oxidative stress are involved in peripheral neuropathy induced by nab-paclitaxel. (a) Western blotting was used to detect the expression of sEH and NF-*κ*B. (b) Effects of TPPU on the levels of inflammatory factors, including TNF-*α*, IL-1*β*, IL-6, IL-4, and IL-10, in the rat spinal cord were determined by ELISA. (c) Oxidative stress was present as the levels and activities of SOD, CAT, MDA, and GSH in the spinal cord. *n* = 6; ^*∗*^*P* < 0.05 compared to the control group, ^#^*P* < 0.05 compared to the nab-P group.

The expressions of sEH and NF-*κ*B were higher in the nab-P group (*P* all <0.001) in comparison with the control group. Compared to the nab-P group, the expression levels of sEH and NF-*κ*B were decreased in the Nab-P + TPPU group (Figure 5(a)) (*P*=0.008, *P*=0.009, respectively). The levels of proinflammatory factors (TNF-*α*, IL-1*β,* and IL-6) were higher and the levels of anti-inflammatory factors (IL-4 and IL-10) were lower in the spinal cords of rats in the nab-P group (*P* all <0.001) in comparison with the control group. Compared to the nab-P group, the levels of TNF-*α*, IL-1*β,* and IL-6 were reduced, and the levels of IL-4 and IL-10 were elevated in the nab-P + TPPU group (Figure 5(b)) (*P* all <0.001). Compared to the control group, the levels of MDA were increased and the levels of SOD, GSH, and CAT were decreased in the nab-P group (*P* all <0.001). Compared to the nab-P group, the levels of MDA were lower, and the levels of SOD, GSH, and CAT were higher in the nab-P + TPPU group (Figure 5(c)) (*P* all <0.001).

## 4. Discussion

The bioactivity of EETs is transient *in vivo* principally due to the rapid metabolism by sEH. Pharmacologic inhibition of sEH has been demonstrated to greatly help in maintaining or elevating EET levels *in vivo* to achieve beneficial effects in neuropathic pain [3, 12]. For the first time, we demonstrated that sEH inhibition with TPPU alleviates mechanical and thermal pain in a model of nab-paclitaxel-induced neuropathic pain. The ameliorative neuropathic pain was associated with improved vascular permeability and decreased spinal cord apoptosis, glial response, and axonal damage. These protective effects of TPPU treatment were related to decreased levels of inflammatory cytokines and oxidative stress by inhibiting the activation of the sEH and NF-*κ*B signalling pathways in the rat spinal cord. These data suggest that sEH inhibition may be a potential therapeutic target of PIPN in rats.

Neurofilaments could encode a neuronal protein and play roles in nerve conduction velocity and axonal transport [13]. NF-L has been regarded as a biomarker of neurologic damage in disease states such as amyotrophic lateral sclerosis, diabetes, and Parkinson's disease and increases with neurotoxic chemotherapy [13, 14]. Serum NF-L and chemotherapy-induced peripheral neuropathy (CIPN) symptoms increased concurrently during taxane treatment [13]. It is possible that NF-L can be used to differentiate women who will develop CIPN during taxane treatment. A previous study found that small interfering RNA-7a could ameliorate neuropathic pain by blocking the activator of the transcription signalling pathway by repressing neurofilament light polypeptide in a spinal nerve ligation rat model [15]. In this study, nab-paclitaxel treatment could increase the expression levels of NF-L, NF-M, and NF-H. sEH inhibition with TPPU could decrease the expression of NF-M, NF-H, and NF-L accompanied by improved mechanical and thermal pain, which indicated that nab-paclitaxel could induce axonal injury in the spinal cord and that neurofilaments might be biomarkers of the development of PIPN.

It has been found that chemotherapeutic agents can accumulate in peripheral nerves and cause neurotoxicity as well as significant nociceptive hypersensitivity [16]. The neurotoxicity caused by chemotherapy drugs might be related to the disruption of the integrity of the BSCB. For example, vincristine treatment affects the integrity of the BSCB by influencing Evans blue extravasation and tight junction disruption in the spinal cord [16]. The endothelial cells that form the BSCB are tight junction-coupled proteins and include claudins, zonula occludens and occludin [17]. After nab-paclitaxel treatment, we detected the downregulation of junction-coupled proteins in the spinal cord tissue. We also found significant increases in EB extravasation and water content. Additionally, the ultrastructure structures surrounding the microvessels were destroyed, with large interstices and oedema. Specifically, TPPU treatment increased the expression levels of the tight junction proteins claudin-5, occludin-1, and ZO-1 in the spinal cord and decreased EB extravasation and water content in neuropathic pain rat models. Therefore, alterations in the permeability of the BSCB after nab-paclitaxel treatment could be related to changes in expression of tight junction protein [17].

EET plays crucial roles in pain, including osteoarthritis knee pain, neuropathic pain, and central poststroke pain [3, 8, 18]. However, EETs are rapidly hydrolysed into dihydroxyeicosatrienoic acid when exposed to sEH. Pharmacologic inhibition of sEH could increase EET levels. The sEH inhibitor TPPU is a novel antihypertensive and anti-atherosclerotic pharmaceutical that attenuated vascular permeability, decreased axonal damage assessed by neurofilament proteins, lowered cell apoptosis, and alleviated glial response in this study. The protective effect of TPPU was related to decreased levels of inflammatory cytokines and oxidative stress by inhibiting activation of the sEH and NF-*κ*B signalling pathways in the rat spinal cord. The activation of the NF-*κ*B signalling pathway is closely correlated with the expansion of inflammatory reactions. The activation of the NF-*κ*B pathway and upregulation of inflammatory factors have been confirmed to aggravate neuropathic pain [19, 20]. In this study, we found the progression of the NF-*κ*B pathway in PIPN and found that TPPU reversed the protein levels of sEH, NF-*κ*B, and downstream inflammatory cytokines and oxidative stress levels which promoted by nab-paclitaxel. TPPU decreased the levels of proinflammatory factors, including TNF-*α*, IL-1*β,* and IL-6, and increased the levels of anti-inflammatory factors, including IL-4 and IL-10, in the spinal cord. Similar to our findings, a study found that TIPE2 reduced the expression of TAK1, thereby inhibiting the activated pathway of NF-*κ*B and further improving sciatic nerve injury-induced neuropathic pain [19]. A growing number of studies suggest that the development and maintenance of neuropathic pain were related to mitochondrial dysfunction and abnormal oxidative stress [21, 22]. In this study, the levels and activities of SOD, CAT, MDA, and GSH were tested, and the results showed that nab-paclitaxel treatment could downregulate antioxidant levels and upregulate oxidative stress. Mitochondrial vacuoles, fractures, swelling, and disordered sparse cristae were also observed after nab-paclitaxel treatment. The sEH inhibitor alleviated mitochondrial injury by downregulating oxidative stress. A previous study found that mitoxantrone reduces neuropathic pain by alleviating mitochondrial dysfunction, inhibiting oxidative stress, and relieving apoptosis [23].

In this study, we also found that the activation of microglial cells and astrocytes was significantly enhanced after nab-paclitaxel treatment. TPPU decreased the activation of glial response. Similar to previous studies, our results also suggested that nab-paclitaxel may promote the activation of glial cells, thereby facilitating the release of inflammatory cytokines and contributing to the aggravation of PIPN [24, 25]. Inflammatory cytokines could activate microglial cells and astrocytes. Microglia and astrocytes are also most affected by a reduction in the inflammatory response [26]. Our previous study also found that inhibition of the Notch pathway could reduce the expression of inflammatory factors accompanied by repressive glia response through the HMGB1/TLR4 signalling pathway in a rat model of nab-paclitaxel-induced peripheral neuropathy [27]. All the results suggested that TPPU may suppress the activation of the glial response through the regulation of inflammatory cytokines.

8–10 weeks-old male rats were used to conduct this research which was consistent with previous studies. Multivariable linear regression analyses which predicted factors of CIPN severity revealed that the male sex was associated with greater CIPN [28]. Thus, the male sex seems to suffer CIPN more easily. Recently, more and more studies were conducted by using both male and female animals simultaneously. However, it is still difficult to determine sex differences from the current literature about CIPN. The 8–10 weeks-old rats correspond to young adults of humans. In several previous studies about CIPN, young adult animals were used as test subjects. Moreover, in the previous study, spared nerve injury of SD rats could perform at 33 days after birth and lead to significant and persistent allodynia with the threshold falling to 55% of control values [29]. It means that mechanical allodynia can be evoked in very young animals with inflammatory pain. However, the median age of many common solid cancers at diagnosis is between 60 to 70 years. The age of young adult rats used in many animal experiments is contradicted by epidemiological evidence of humans [30]. Aging also could change the function and structure of the peripheral nerves and inhibit compensatory reinnervation [31]. Thus, in order to gain more generalizability of findings from animal studies of CIPN, the inclusion of groups of older animals should be considered [31].

In conclusion, our current study shows that sEH inhibition by TPPU could alleviate PIPN by decreasing cell apoptosis, glial activation, and axonal injury and ameliorating the integrity of the blood-spinal cord barrier through the sEH/NF-*κ*B signalling pathway and downstream inflammatory cytokines and oxidative stress. These results suggest that the sEH inhibitor TPPU can serve as a potential drug target for the treatment of PINP.

## 5. Disclosure

This article is a follow-up study cited as Wei X., Zhou Y. Q., Ma L., Li W. M., Shan C. Y., Zhang S. Q., and Zhao Y. L. Inhibition of Notch Pathway Alleviates Nab-Paclitaxel-Induced Peripheral Neuropathic Pain in Rats via Suppressing HMGB1/TLR4 Signaling in Spinal Cord, 09 September, 2022, PREPRINT (Version 1) available at Research Square (https://doi.org/10.21203/rs.3.rs-2022168/v1).

## Data Availability

Some or all data, models, or code generated or used during the study are available in a repository or online in accordance with funder data retention policies (provide full citations that include URLs or DOIs.).

## Supplementary Materials

Supplementary Materials The supplementary materials include the raw data of the article, ARRIVE Full checklist, and the CONSORT checklist.
