# Supplementary material for: Soluble Epoxide Hydrolase Inhibitor TPPU Alleviates Nab-Paclitaxel-Induced Peripheral Neuropathic Pain via Suppressing NF-κB Signalling in the Spinal Cord of a Rat
Source: Pain Res Manag. 2023 Feb 8;2023:9058774. doi: 10.1155/2023/9058774 (PMC9931472; doi:10.1155/2023/9058774)
Supplement: Supplementary Materials — The supplementary materials include the raw data of the article, ARRIVE Full checklist, and the CONSORT checklist. [file 9058774.f1.zip › Author Checklist - Full_wrapper_wrapper-2.pdf]

NOTE: Please save this file locally before filling in the table, DO NOT work on the file within your internet browser as changes will not be saved. Adobe Acrobat Reader (available free here) is recommended for completion.

删除标识 | 福昕编辑器

ARRIVE

The ARRIVE guidelines 2.0: author checklist

The ARRIVE Essential 10

These items are the basic minimum to include in a manuscript. Without this information, readers and reviewers cannot assess the reliability of the findings.

Item		Recommendation	Section/line number, or reason for not reporting
**Study design**	1	For each experiment, provide brief details of study design including:	
		a. The groups being compared, including control groups. If no control group has been used, the rationale should be stated.	2.3 Groups and drug administration section/line 2-7
		b. The experimental unit (e.g. a single animal, litter, or cage of animals).	2.3 Groups and drug administration section/line 2-7
**Sample size**	2	a. Specify the exact number of experimental units allocated to each group, and the total number in each experiment. Also indicate the total number of animals used.	2.3 Groups and drug administration section/line 1-5
		b. Explain how the sample size was decided. Provide details of any *a priori* sample size calculation, if done.	2.3 Groups and drug administration section/line 1-2
**Inclusion and exclusion criteria**	3	a. Describe any criteria used for including and excluding animals (or experimental units) during the experiment, and data points during the analysis. Specify if these criteria were established *a priori*. If no criteria were set, state this explicitly.	No animals were included and excluded during the experiment. The animal model was stable. the species, strain and substrain, sex, age and developmental stage of rats were consistent
		b. For each experimental group, report any animals, experimental units or data points not included in the analysis and explain why. If there were no exclusions, state so.	n=6 in each experimental group, and each experiment was repeated 3 times. all the results were recorded and analyzed
		c. For each analysis, report the exact value of *n* in each experimental group.	in all 3. Results section, the exact value of n was present in each subunit of all results.
**Randomisation**	4	a. State whether randomisation was used to allocate experimental units to control and treatment groups. If done, provide the method used to generate the randomisation sequence.	2.3 Groups and drug administration section/line 1
		b. Describe the strategy used to minimise potential confounders such as the order of treatments and measurements, or animal/cage location. If confounders were not controlled, state this explicitly.	2.3 Groups and drug administration section/line 14
**Blinding**	5	Describe who was aware of the group allocation at the different stages of the experiment (during the allocation, the conduct of the experiment, the outcome assessment, and the data analysis).	2.3 Groups and drug administration section/line 16-17
**Outcome measures**	6	a. Clearly define all outcome measures assessed (e.g. cell death, molecular markers, or behavioural changes).	in all 2. Materials and Methods section
		b. For hypothesis-testing studies, specify the primary outcome measure, i.e. the outcome measure that was used to determine the sample size.	Not relevant to this study
**Statistical methods**	7	a. Provide details of the statistical methods used for each analysis, including software used.	2.15 Statistical Analysis section/line 1-4
		b. Describe any methods used to assess whether the data met the assumptions of the statistical approach, and what was done if the assumptions were not met.	2.15 Statistical Analysis section/line 1-4
**Experimental animals**	8	a. Provide species-appropriate details of the animals used, including species, strain and substrain, sex, age or developmental stage, and, if relevant, weight.	2.1 Experimental animals and ethical approval section/line 1-2
		b. Provide further relevant information on the provenance of animals, health/immune status, genetic modification status, genotype, and any previous procedures.	all the information is not relevant to this study
**Experimental procedures**	9	For each experimental group, including controls, describe the procedures in enough detail to allow others to replicate them, including:	
		a. What was done, how it was done and what was used.	in all 2. Materials and Methods section
		b. When and how often.	in all 2. Materials and Methods section
		c. Where (including detail of any acclimatisation periods).	in all 2. Materials and Methods section
		d. Why (provide rationale for procedures).	in all 2. Materials and Methods section
**Results**	10	For each experiment conducted, including independent replications, report:	
		a. Summary/descriptive statistics for each experimental group, with a measure of variability where applicable (e.g. mean and SD, or median and range).	in all 3. Result section including figure legend
		b. If applicable, the effect size with a confidence interval.	in all 3. Result section including figure legend

福昕PDF编辑器

The Recommended Set

These items complement the Essential 10 and add important context to the study. Reporting the items in both sets represents best practice.

Item		Recommendation	Section/line number, or reason for not reporting
**Abstract**	11	Provide an accurate summary of the research objectives, animal species, strain and sex, key methods, principal findings, and study conclusions.	abstract section/line 1-14
**Background**	12	a. Include sufficient scientific background to understand the rationale and context for the study, and explain the experimental approach.	1. Introduction section/line 1-16
		b. Explain how the animal species and model used address the scientific objectives and, where appropriate, the relevance to human biology.	1. Introduction section/line 17-22
**Objectives**	13	Clearly describe the research question, research objectives and, where appropriate, specific hypotheses being tested.	1. Introduction section/line 17-22
**Ethical statement**	14	Provide the name of the ethical review committee or equivalent that has approved the use of animals in this study, and any relevant licence or protocol numbers (if applicable). If ethical approval was not sought or granted, provide a justification.	2.1 Experimental animals and ethical approval section/line 1-6
**Housing and husbandry**	15	Provide details of housing and husbandry conditions, including any environmental enrichment.	2.1 Experimental animals and ethical approval section/line 6-8
**Animal care and monitoring**	16	a. Describe any interventions or steps taken in the experimental protocols to reduce pain, suffering and distress.	2.2 Establishment of a paclitaxel-induced peripheral neuropathy model section/line 5-7
		b. Report any expected or unexpected adverse events.	none
		c. Describe the humane endpoints established for the study, the signs that were monitored and the frequency of monitoring. If the study did not have humane endpoints, state this.	2.2 Establishment of a paclitaxel-induced peripheral neuropathy model section/line 5-7
**Interpretation/ scientific implications**	17	a. Interpret the results, taking into account the study objectives and hypotheses, current theory and other relevant studies in the literature.	discussion section/line 1-74
		b. Comment on the study limitations including potential sources of bias, limitations of the animal model, and imprecision associated with the results.	discussion section/line 75-90
**Generalisability/ translation**	18	Comment on whether, and how, the findings of this study are likely to generalise to other species or experimental conditions, including any relevance to human biology (where appropriate).	discussion section/line 75-90
**Protocol registration**	19	Provide a statement indicating whether a protocol (including the research question, key design features, and analysis plan) was prepared before the study, and if and where this protocol was registered.	this study was not involved in human and there is a licence number in the article
**Data access**	20	Provide a statement describing if and where study data are available.	Data Availability section/line 1-2
**Declaration of interests**	21	a. Declare any potential conflicts of interest, including financial and non-financial. If none exist, this should be stated.	Competing Interests section/line 1
		b. List all funding sources (including grant identifier) and the role of the funder(s) in the design, analysis and reporting of the study.	Funding Information section/line 1-2
