# Supplementary figures and images for: Soluble Epoxide Hydrolase Inhibitor TPPU Alleviates Nab-Paclitaxel-Induced Peripheral Neuropathic Pain via Suppressing NF-κB Signalling in the Spinal Cord of a Rat
Source: Pain Res Manag. 2023 Feb 8;2023:9058774. doi: 10.1155/2023/9058774 (PMC9931472; doi:10.1155/2023/9058774)

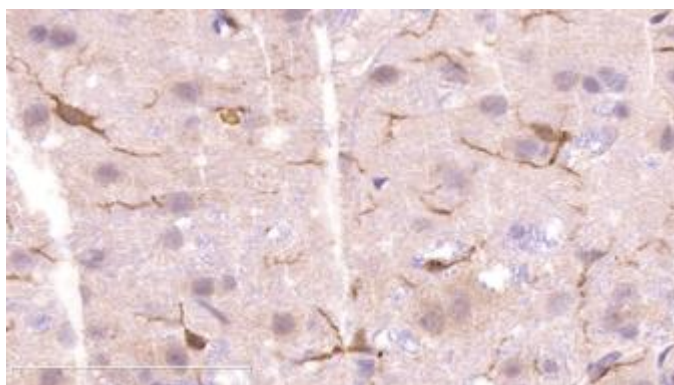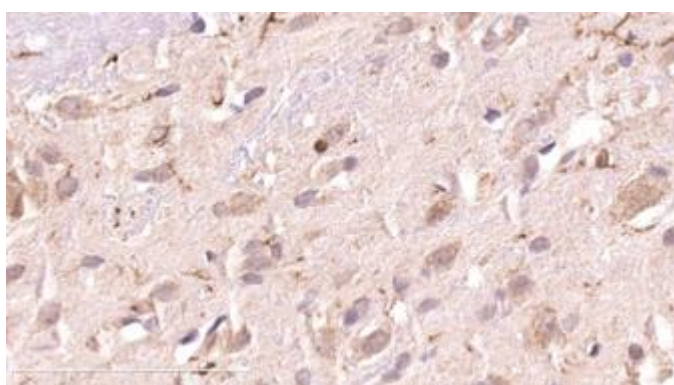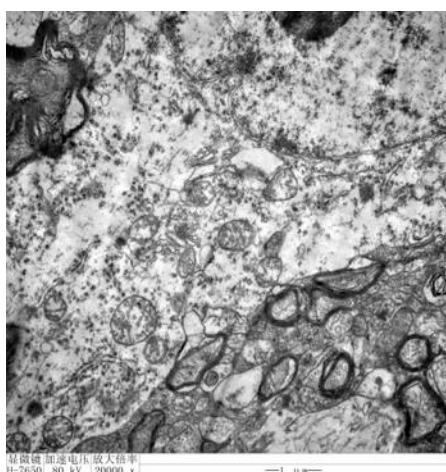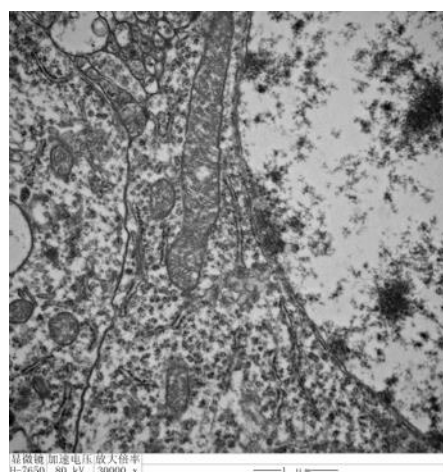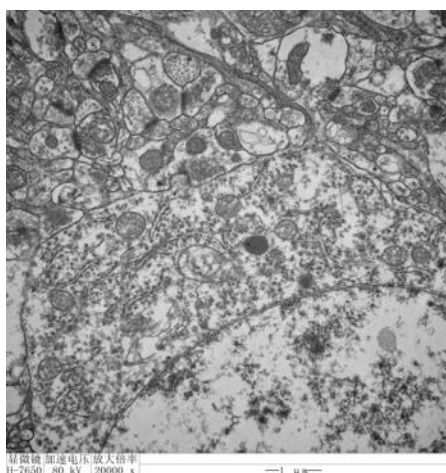

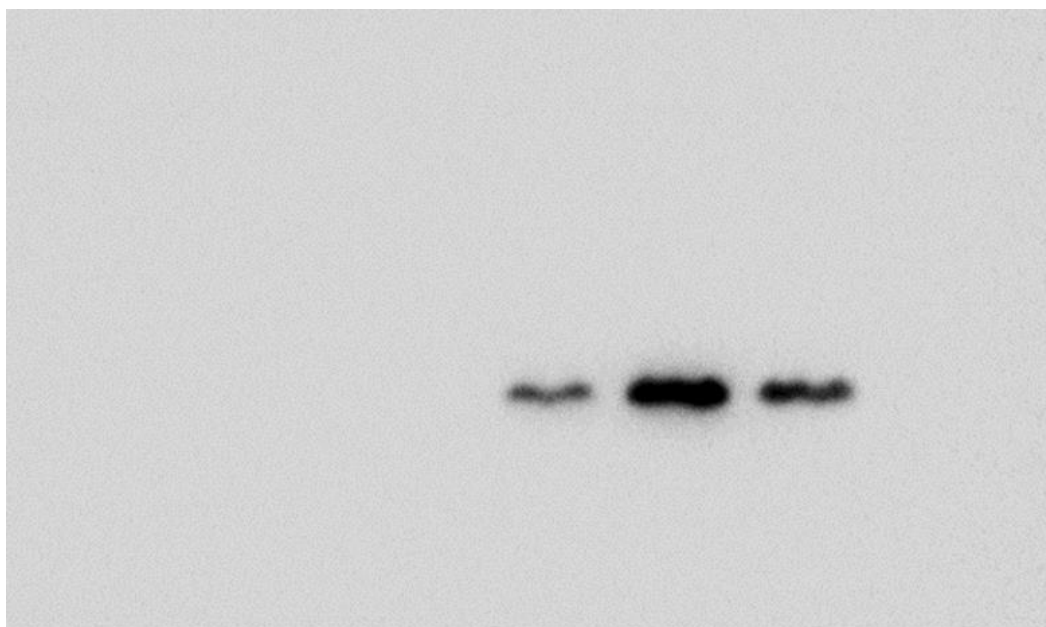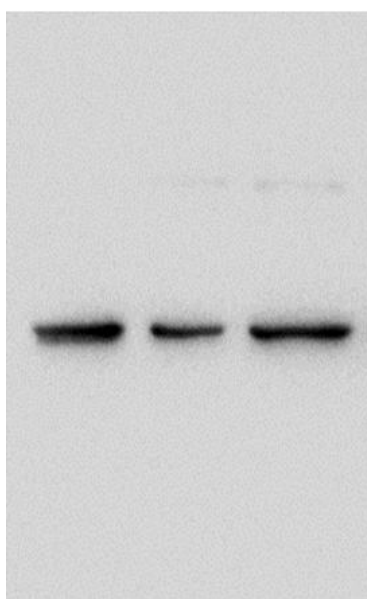

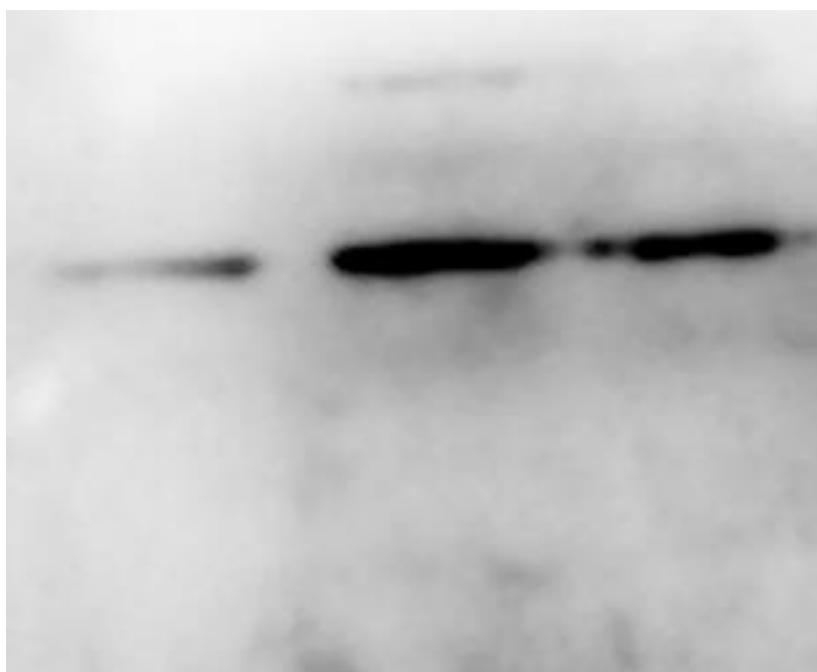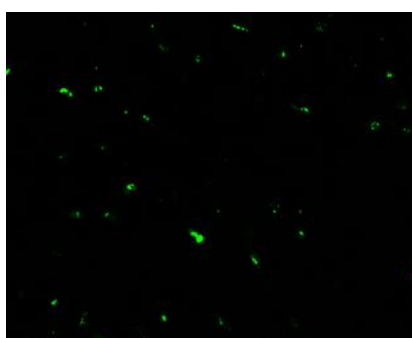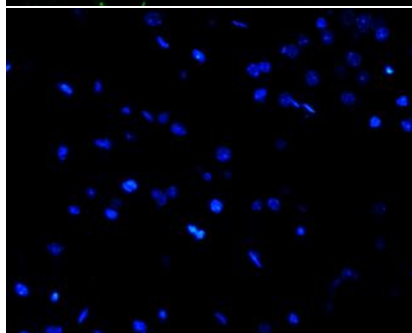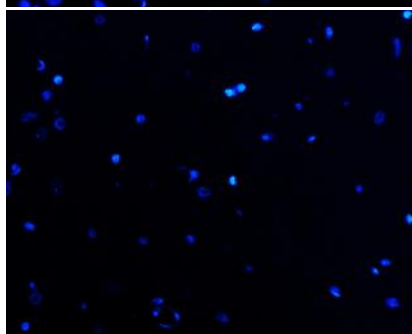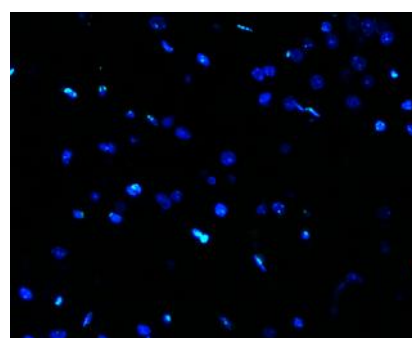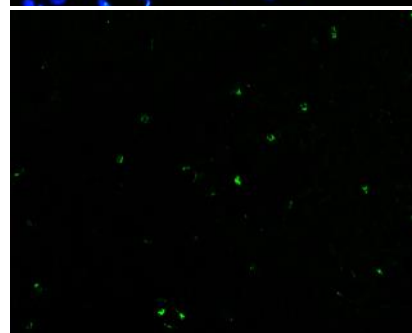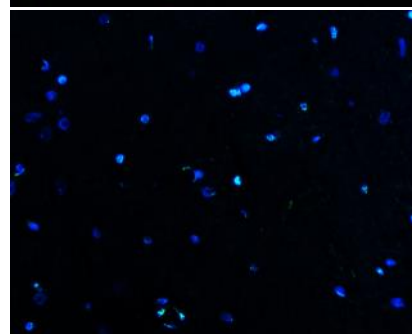

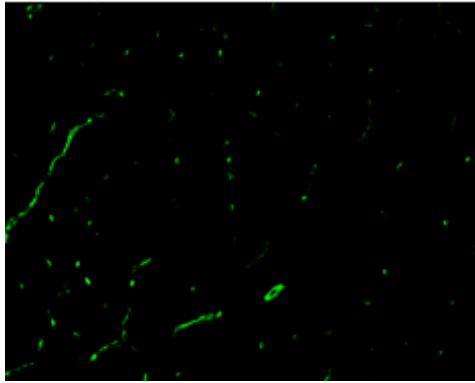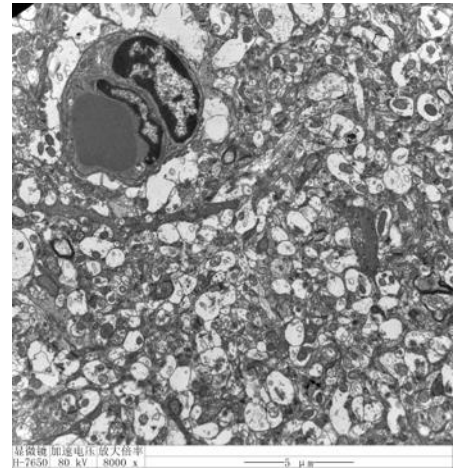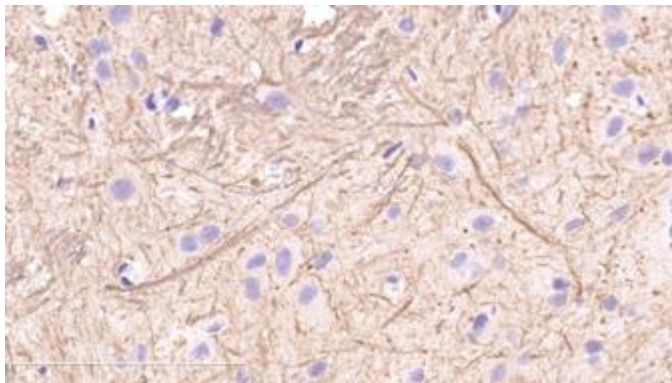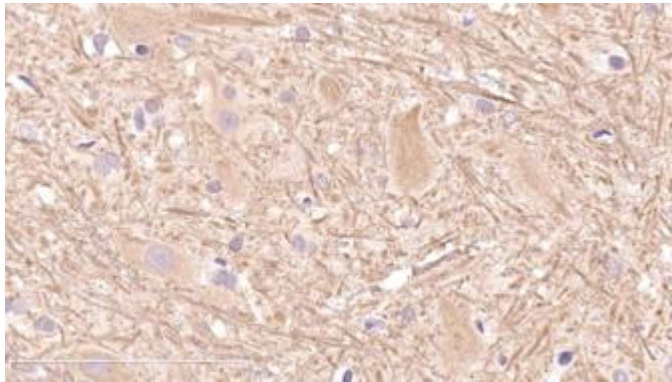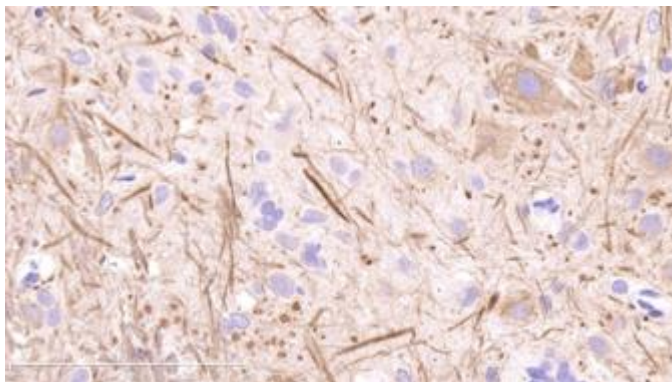

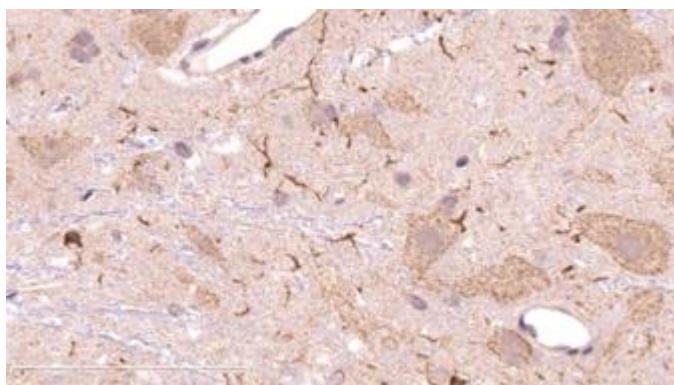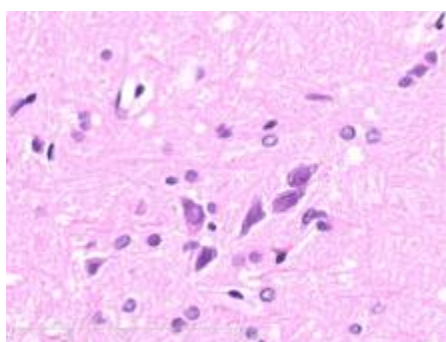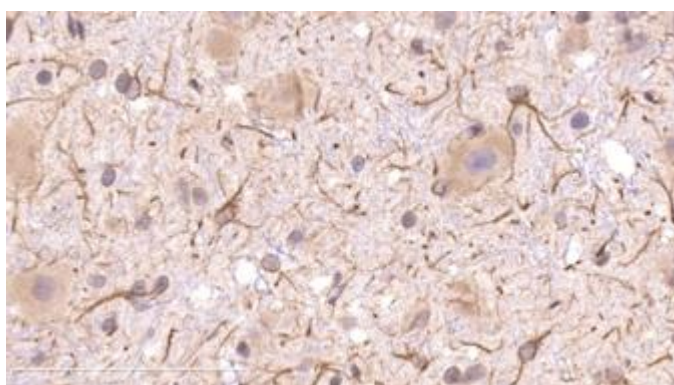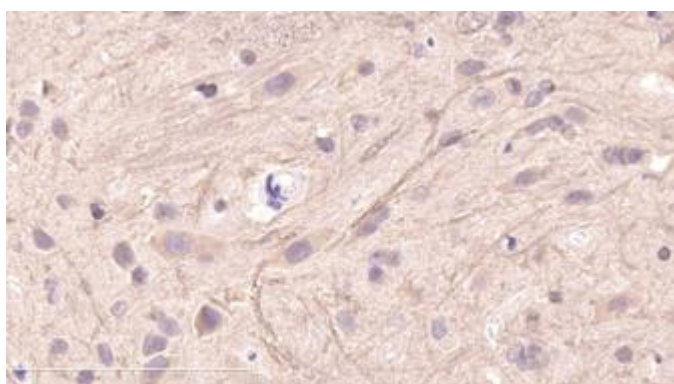

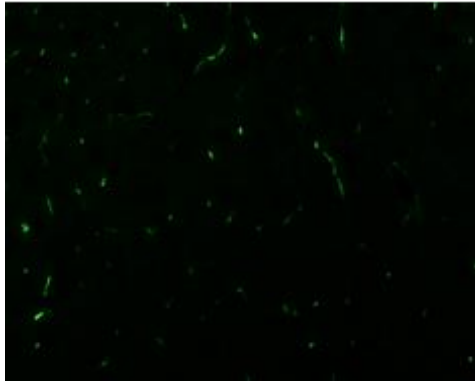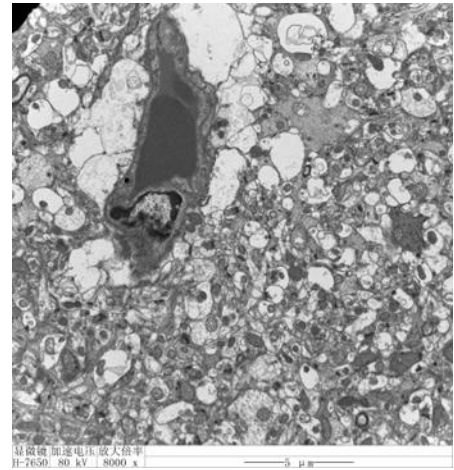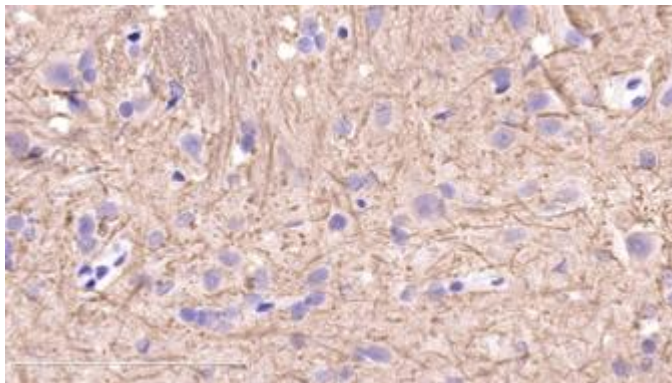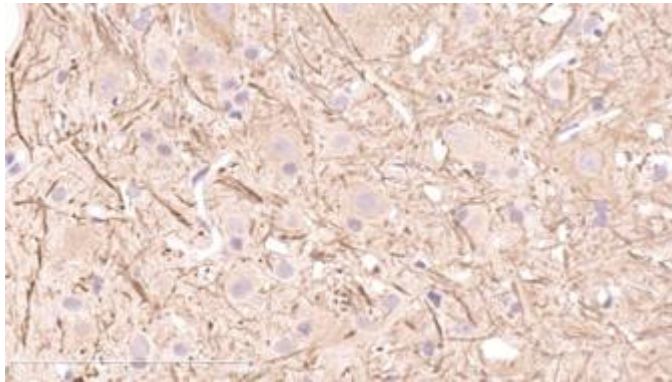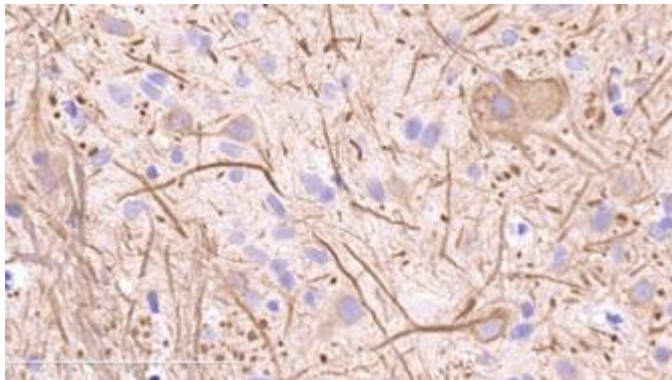

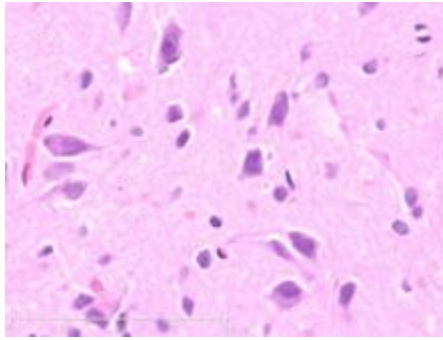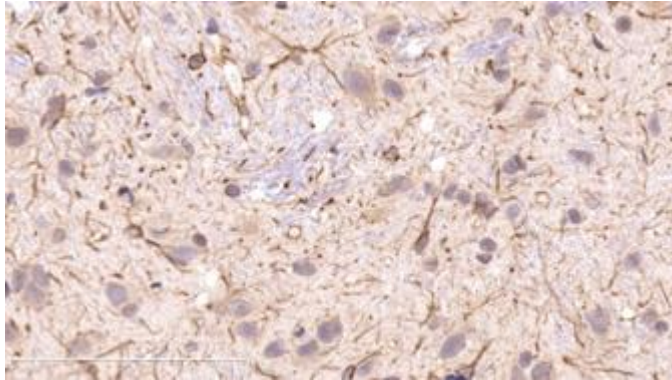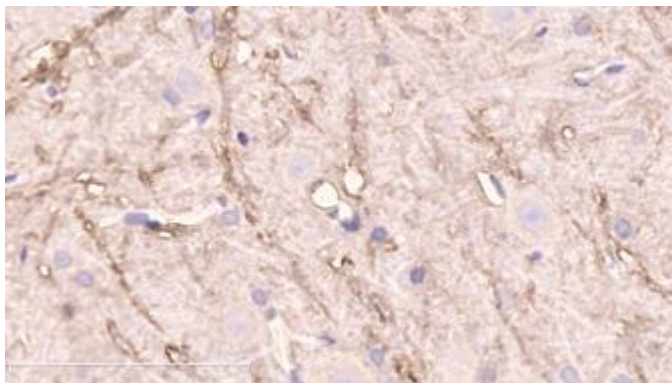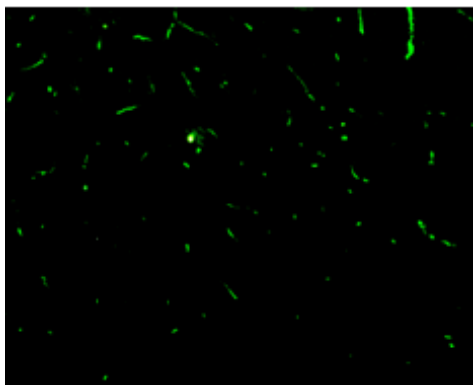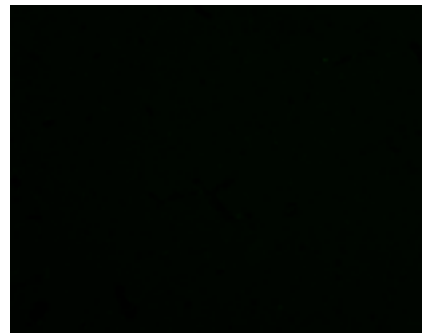

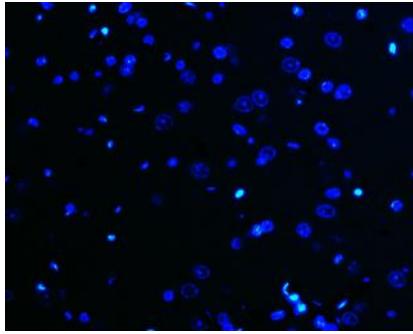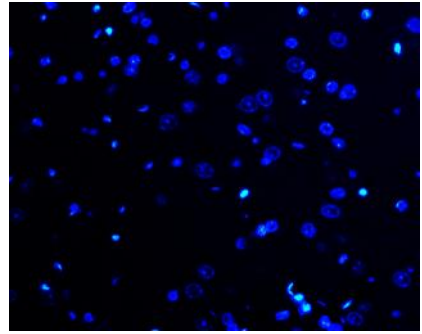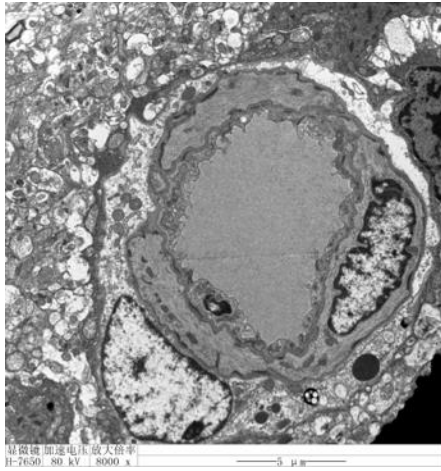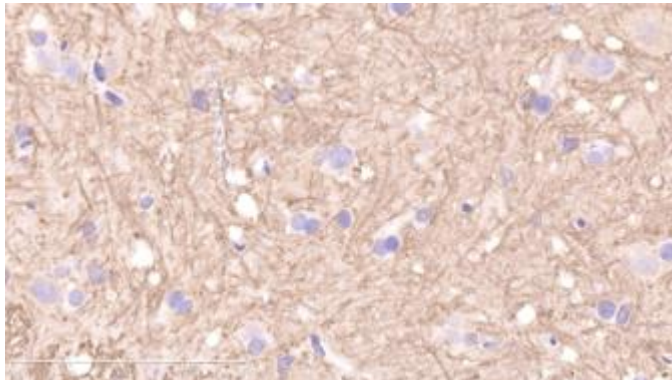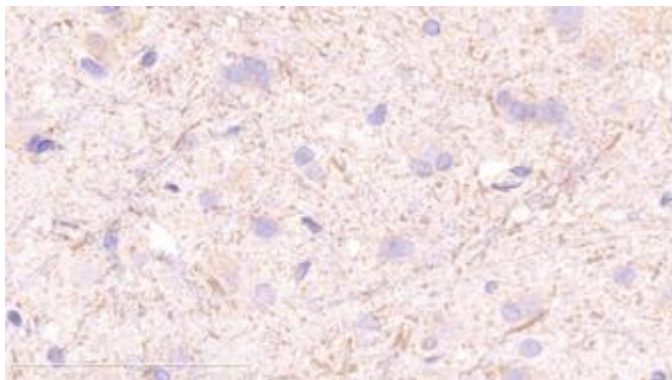

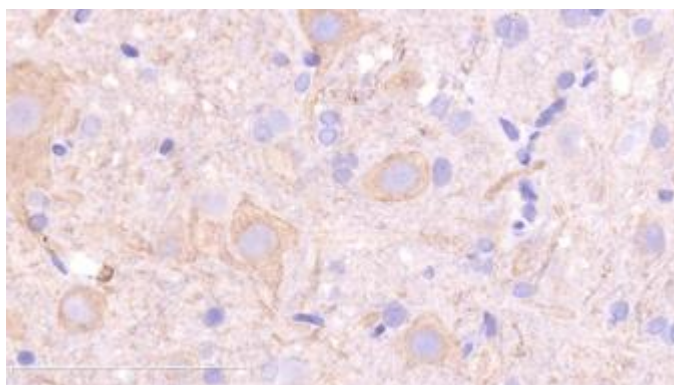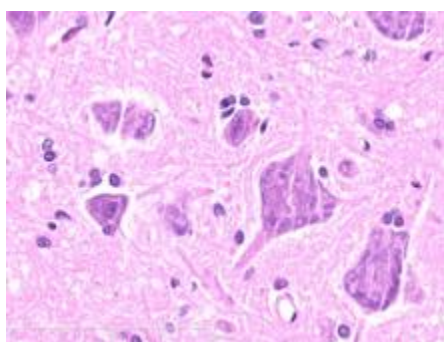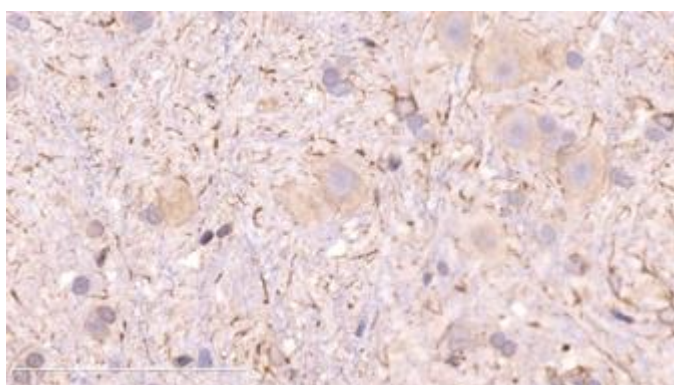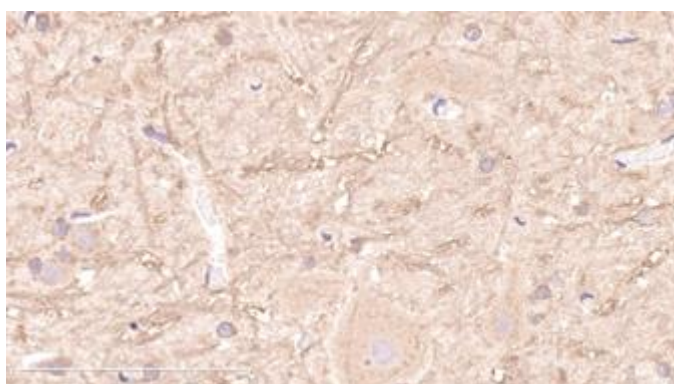

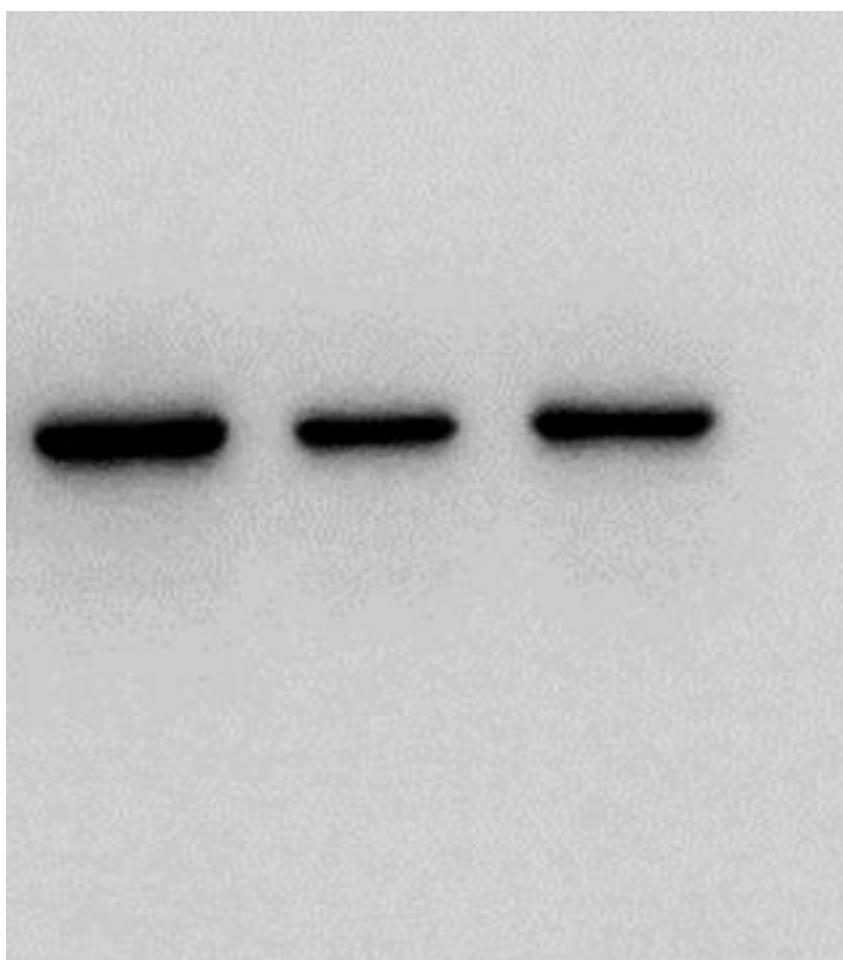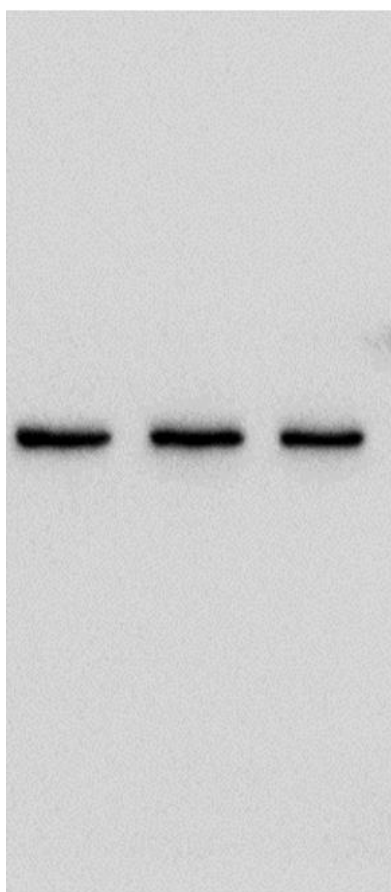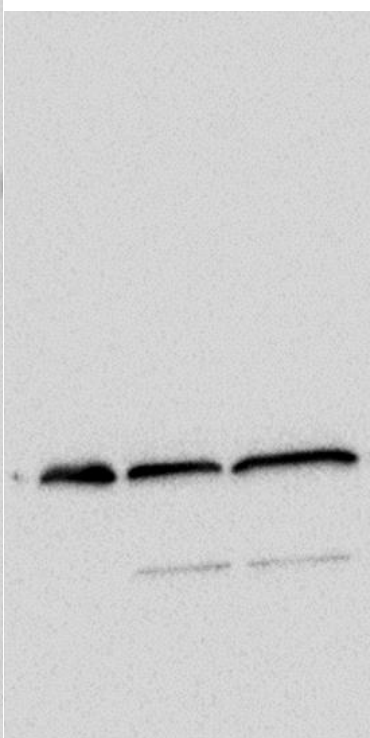

Supplement: Supplementary Materials — The supplementary materials include the raw data of the article, ARRIVE Full checklist, and the CONSORT checklist. [file 9058774.f1.zip › raw data-figure (1).pdf]
